# Differential response to scrambler therapy by neuropathic pain phenotypes

**DOI:** 10.1038/s41598-021-89667-6

**Published:** 2021-05-12

**Authors:** Young Gi Min, Hyun Seok Baek, Kyoung-Min Lee, Yoon-Ho Hong

**Affiliations:** 1 Department of Neurology, Seoul National University Hospital, Seoul, South Korea; 2 Department of Neurology, Seoul National University College of Medicine, Seoul, South Korea; 3 Department of Neurology, Neuroscience Research Insitute, Seoul National University Medical Research Council, Seoul Metropolitan Government-Seoul National University Boramae Medical Center, 20 Boramaero-5-Gil, Dongjak-Gu, Seoul, 07061 South Korea

**Keywords:** Neurology, Neuropathic pain, Peripheral neuropathies, Spinal cord diseases

## Abstract

Scrambler therapy is a noninvasive electroanalgesia technique designed to remodulate the pain system. Despite growing evidence of its efficacy in patients with neuropathic pain, little is known about the clinical factors associated with treatment outcome. We conducted a prospective, open-label, single-arm trial to assess the efficacy and safety of scrambler therapy in patients with chronic neuropathic pain of various etiologies. A post-hoc analysis was performed to investigate whether cluster analysis of the Neuropathic Pain Symptom Inventory (NPSI) profiles could identify a subgroup of patients regarding neuropathic pain phenotype and treatment outcome. Scrambler therapy resulted in a significant decrease in the pain numerical rating scale (NRS) score over 2 weeks of treatment (least squares mean of percentage change from baseline, − 15%; 95% CI − 28% to − 2.4%; p < 0.001). The mean score of Brief Pain Inventory (BPI) interference subdimension was also significantly improved (p = 0.022), while the BPI pain composite score was not. Hierarchical clustering based on the NPSI profiles partitioned the patients into 3 clusters with distinct neuropathic pain phenotypes. Linear mixed-effects model analyses revealed differential response to scrambler therapy across clusters (p = 0.003, pain NRS; p = 0.072, BPI interference subdimension). Treatment response to scrambler therapy appears different depending on the neuropathic pain phenotypes, with more favorable outcomes in patients with preferentially paroxysmal pain rather than persistent pain. Further studies are warranted to confirm that capturing neuropathic pain phenotypes can optimize the use of scrambler therapy.

## Introduction

Neuropathic pain is pain caused by a lesion or disease of the peripheral and/or central somatosensory nervous system^1^. Comprising a wide range of heterogeneous conditions, it is highly prevalent, affecting up to 10% of the general population^2^. Neuropathic pain can have a profound negative impact on the quality of life of affected patients, and it can also have high socioeconomic costs^3,4^. Pharmacologic treatment with analgesics such as tricyclic antidepressants, serotonin-noradrenaline reuptake inhibitors, and gabapentinoids is typically the first step in managing neuropathic pain^5,6^. However, pharmacotherapy alone may be limited with regard to efficacy and tolerance, with less than one-third of patients experiencing 50% or greater pain relief^5–9^.

Scrambler therapy is a noninvasive electroanalgesia technique that utilizes transcutaneous electrical stimulation to reorganize maladaptive pain signaling pathways^10,11^. The active principle with scrambler therapy is that artificial strings of action potentials calibrated to synchronize C-fiber surface receptors may replace endogenous pain information with synthetic "non-pain" information^11^. It is based on a different theoretical mechanism than the traditional gate control theory in which the electric stimulation of A-beta fibers is delivered to block pain signals and produce an analgesic effect. Since the first preliminary result was reported in patients with drug-resistant visceral cancer pain in 2003^12^, more than 20 studies of varying scientific quality followed in a variety of chronic pain conditions, including neuropathic pain^10,11,13–16^. Despite substantial effects in some studies, however, the treatment outcomes seem to be highly variable across studies, with little known about clinical factors associated with the variability in treatment response^17,18^.

A therapeutic approach based on pathophysiologic mechanisms of neuropathic pain could help identify patients who are more likely to benefit from a particular treatment^19–25^. Different pathophysiologic mechanisms may manifest as various pain-related sensory symptoms and signs^8,19,21,26,27^. While it is still challenging to unravel complex mechanism-phenotype relationships, it would be useful to analyze the pattern of pain-related symptom profiles as a whole, rather than individual symptoms and signs. Using the validated symptom-based questionnaires or quantitative sensory testing (QST), researchers have identified subgroups of patients characterized by distinct neuropathic pain phenotypes, which might be related to specific pathophysiologic mechanisms^21,26^. Furthermore, several studies have shown that particular drugs have better efficacy in subgroups of patients with particular neuropathic pain phenotypes than others, possibly because the pathophysiologic mechanisms engaged are relevant to the drug targets^22,28–31^.

We conducted a prospective, open-label, single-arm trial to evaluate the efficacy and safety of scrambler therapy in patients with chronic drug-resistant neuropathic pain of various etiologies. A post-hoc cluster analysis was performed to investigate whether clustering based on the pain-related symptom profiles (NPSI) can identify distinct subgroups of patients with regard to neuropathic pain phenotype and response to scrambler therapy.

## Methods

### Study design

This study was an open-label, single-arm trial to evaluate the efficacy and safety of scrambler therapy in patients with chronic drug-resistant neuropathic pain at the Seoul National University Seoul Metropolitan Government Boramae Medical Center in Seoul, South Korea.

### Participants

Eligible patients were adults aged 80 years or younger who were seen in the outpatient clinic and who had chronic drug-resistant neuropathic pain caused by peripheral nerve or spinal cord lesions. Pain lasting for more than 6 months was rated as 4 or higher on an 11-point numerical rating scale (NRS) despite the active use of standard pain medications, including anticonvulsants, antidepressants, or opioids. The medications for pain treatment were not changed or adjusted within the month prior to the initiation of scrambler therapy. The neuropathic nature of the pain was confirmed on the painDETECT questionnaire, as reflected by a total score of 13 or higher. Exclusion criteria were as follows: (1) pregnancy or breast feeding; (2) coronary stents or metal implants, including pacemakers, automatic defibrillators, aneurysm clips, or metallic artificial joints; (3) a history of epilepsy or traumatic brain injury; (4) a history of myocardial infarction or ischemic heart disease within the past 6 months; (5) skin conditions for which the attachment of electrodes is not appropriate; and (6) cognitive impairment or mental incompetence.

### Intervention

The study procedures are summarized in Supplementary Figure 1. With informed consent, the eligible subjects were screened to verify the neuropathic nature of their pain using the painDETECT questionnaire. The Neuropathic Pain Symptom Inventory (NPSI) and Brief Pain Inventory-Short Form (BPI-SF) were administered to the included participants before the initiation of the treatment sessions. Throughout 2 weeks (10 business days) of scrambler therapy, participants were asked to rate the overall intensity of their pain on a daily basis immediately prior to receiving scrambler therapy. The BPI-SF was re-administered after 2 weeks of treatment sessions and then again after 2 weeks of follow-up.

Scrambler therapy was administered according to the established protocol (GEOMC, model NPC3, Seoul, South Korea) by a trained technician under the supervision of the principal investigator^11^. Briefly, a pair of electrodes was attached such as to surround the surface area of the pain, within the same dermatome whenever possible. The stimulation current intensity gradually increased to a degree that the patient could still tolerate (maximum 5.5 mA). If the patient felt a constant burn, sting, or discomfort, the electrodes were repositioned and the procedure was resumed from the beginning. The optimal position of the electrodes was guided by the analgesic response of the patient. Once the position of the electrodes was established, stimulation of the maximum tolerable intensity was administered for 30 min. Depending on the number and size of the surface pain area, additional pairs of electrodes were applied as appropriate, with up to 3 channels in total. The treatment sessions were provided daily over 2 weeks with 2-day interruptions.

### Outcomes

The painDETECT questionnaire is a screening instrument that was originally developed to identify neuropathic components in patients with chronic low back pain^32^. It consists of 7 pain-related sensory symptom items (graded from 0 = never to 5 = strongly), 1 temporal item on pain-course pattern (graded from − 1 to + 1), and 1 spatial item on pain radiation (graded 0 = no radiation or + 2 = radiating pain). A total score ranging from − 1 to 38 can be calculated from the 9 items, with scores ≥ 13 indicating possible or probable neuropathic pain.

The NPSI is a self-administered questionnaire consisting of 10 neuropathic pain-related symptom descriptors on an 11-point NRS. It is comprised of 5 subscales, representing distinct dimensions of neuropathic pain: superficial spontaneous pain (burning), deep spontaneous pain (pressing, squeezing), paroxysmal pain (electric shocks, stabbing), evoked pain (evoked by brushing, pressure, cold stimuli), and paresthesia/dysesthesia (pins and needles, tingling)^33^. There are two additional items assessing the temporal pattern of pain, that is, the duration of spontaneous ongoing pain and the frequency of pain attacks according to 5-point categories.

The BPI-SF is a 9-item questionnaire that evaluates the severity of pain and its impact on daily functioning^34^. Patients report their pain in terms of the worst, least, average, and current intensity on an 11-point scale. In a similar manner, the degree of interference with general activity, mood, walking, work, relationships with others, sleep, and enjoyment of life are assessed.

The primary outcome was the percentage change from baseline in the pain NRS scores over the course of 10 treatment sessions. The secondary outcomes were the absolute changes from baseline at 2 and 4 weeks in the mean scores of the BPI subdimensions (pain and interference). The primary and secondary outcomes were analyzed using a linear mixed-effects model, as described below (statistical analysis). Missing data were imputed by the principle of the last observation carried forward.

### Cluster analysis

To partition the patients into distinct subgroups based on the similarities of the 10 NPSI item score profiles, we used an agglomerative hierarchical clustering approach. In this approach, each patient is treated as their own cluster in the beginning, and pairs of clusters are sequentially combined into larger clusters based on the dissimilarity measured between the clusters. The 10 NPSI item scores were normalized to calculate the Pearson correlation distance (dissimilarity measure), and the average inter-cluster distance was used to define the distance between clusters (average linkage)^35^.

To estimate the optimal number of clusters, we used the NbClust R package, in which a variety of internal validity measures such as CH index, Duda index, and Gamma (30 indices in total) are implemented to evaluate how well the clustering results fit the data set. The best number of clusters was determined according to the majority rule, in which the number of clusters proposed by the most indices is considered the optimal number of clusters.

To further assess the stability of the cluster membership assignments, we performed Monti consensus clustering using the ConsensusClusterPlus R package^36,37^. The consensus clustering method involves iterative subsampling and clustering of a set of items (patients). To assess the tendency of items to co-cluster across cluster sets generated in randomly selected subsamples, it calculates pairwise consensus values, reflecting the proportion of 2 items occurring in the same cluster to the number of times they occurred in the same subsample. This is then stored in a symmetrical consensus matrix for each number of the cluster^37^.

### Statistical analysis

Baseline characteristics were compared between patient clusters using Fisher’s exact tests and ANOVA, as appropriate. The primary and secondary outcomes were assessed using a linear mixed-effects model with fixed effects for time, cluster, etiology, sex, use of anticonvulsants, and the interaction terms (time × cluster and time × etiology) and random intercepts for individual participants. All statistical analyses were performed using the statistical software R (version 3.5). Tests with a 2-sided significance level of < 5% were considered statistically significant.

### Ethics approval

This study was approved by the local institutional review board and conducted in accordance with the International Conference on Harmonization Good Clinical Practice Guidelines and the Declaration of Helsinki. All participants provided written informed consent before the study.

## Results

### Clustering

**Figure 1:**
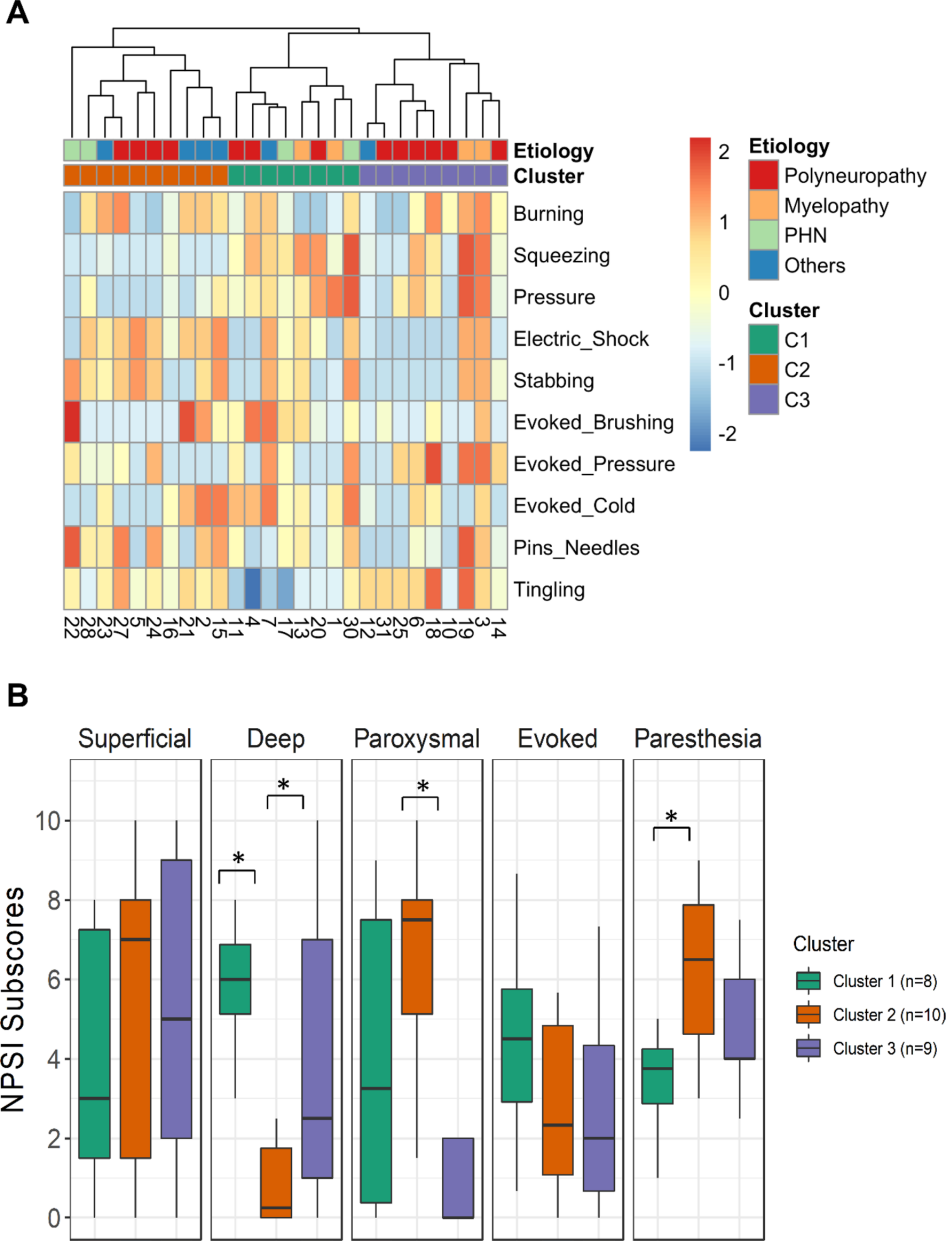
Heatmap Display of the NPSI Profiles (**A**) and Comparisons of the NPSI Subscores by Cluster (**B**). Patient cluster and etiology group membership are indicated by row annotations of different colors above the heatmap and below the dendrogram. Patient clustering was performed on normalized data of the 10 NPSI item scores using Pearson correlation distance and average linkage. NPSI, Neuropathic Pain Symptom Inventory. * indicates statistical significance (p < 0.05, ANOVA).

A total of 31 patients participated in this study, but 4 withdrew their consent prior to the start of scrambler therapy. Of the 27 remaining patients, 25 completed the study: 1 dropped out because of an adverse event on day 8 (contact dermatitis), and the other withdrew consent on day 10 (Supplementary Figure 2). Hierarchical clustering was performed to partition the 27 patients into distinct subgroups such that the patients with similar profiles according to the 10 NPSI item scores were clustered into the same subgroup. The optimal number of clusters was determined to be 3 according to the majority rule, as the most internal validity indices implemented in NbClust R package (9 of 27) indicated it as the best number of clusters (Supplementary Table 1). The consensus clustering results further confirmed the stability of the cluster membership assignment at K = 3 (Supplementary Figure 3). The hierarchical clustering results and heatmap representation of the sensory profiles are shown, along with the comparisons of the scores for the 5 NPSI dimensions across clusters, in Fig. 1.

### Baseline characteristics of patients and comparisons between clusters

The clinical features of the participants are summarized in Table 1. The mean age was 61 years (SD 8.9 years), and the median time from symptom onset to scrambler therapy was 23 months (IQR 13–57 months). The most common etiology was polyneuropathy (n = 13), followed by postherpetic neuralgia (n = 4) and myelopathy (n = 4). Other causes were spondylotic radiculopathy (n = 2), traumatic nerve injury (n = 2), brachial plexopathy (n = 1), and subacute combined degeneration (n = 1). The mean painDETECT total score was 18 (SD 4.7), thereby confirming a neuropathic component of the pain. Pain was mostly severe, with 74% (20 of 27 patients) reporting ratings of 7–10 on the 11-point pain NRS. Over half of the participants (55%, 15 of 27) were on combination therapy with 2 or more drugs of different classes: anticonvulsants in 25 participants, antidepressants in 14, and opioids in 4.

**Table 1:** Baseline characteristics.

	Total (n = 27)	Cluster 1 (n = 8)	Cluster 2 (n = 10)	Cluster 3 (n = 9)	P-value
Sex, female	11 (41%)	6 (75%)	3 (30%)	2 (22%)	0.076
Age, y	61 (8.9)	65 (10.1)	61.2 (10)	58.4 (5.8)	0.33
**Etiology**					0.26
Polyneuropathy^a^	13	3	4	6	
Myelopathy^b^	4	2	0	2	
PHN	4	2	2	0	
Others^c^	6	1	4	1	
Duration, month	23 (13–57)	27 (14–46)	21 (15–62)	15 (11–52)	0.73
painDETECT total score	18 (4.7)	19 (6.5)	18 (3.8)	16 (3.9)	0.52
NPSI total score	41 (20)	45 (20)	41 (12)	37 (28)	0.7
**BPI subdimension scores**					
Pain	5.9 (1.9)	4.6 (1.3)	5.5 (1.6)	7.5 (1.5)	< 0.01
Interference	5.3 (2.3)	4.0 (2.2)	4.7 (2.1)	7.0 (1.6)	< 0.01
**NRS pain score**					0.21
Mild (0–3)	0	0	0	0	
Moderate (4–6)	7	4	2	1	
Severe (7–10)	20	4	8	8	
**Pain medications**					
Anticonvulsants	25	8	9	8	
Antidepressants	14	6	3	5	
Opioids	5	2	2	1	
**Number of poly-drug users**	15	6	5	4	0.42
2 drugs	10	4	3	3	
3 drugs	2	0	2	0	
4 drugs	3	2	0	1	

Data are expressed as number (%), mean (SD) and median (IQR) as appropriate. P-values are obtained using Fisher’s exact test for the categorical variables, and ANOVA for continuous variables.

PHN, Postherpetic Neuralgia; NPSI, Neuropathic Pain Symptom Inventory; NRS, Numerical Rating Scale.

^a^The causes of polyneuropathy include drugs (5 chemotherapeutic agents, 4 isoniazid, 1 linezolid), diabetes mellitus (1), Charcot-Marie-Tooth disease (1), and Guillain–Barre syndrome (1).

^b^The causes of myelopathy include idiopathic transverse myelitis (2), spinal cord infarct (1), and cavernous hemangioma in spinal cord (1).

^c^Others include spondylotic radiculopathy (2), traumatic nerve injury (2), brachial plexopathy (1 schwannoma), and subacute combined degeneration (1).

Comparisons of baseline features across clusters showed that the proportion of females was higher in Cluster 1 (p = 0.076), and the mean scores of the BPI pain and interference subdimensions were significantly higher in Cluster 3 (p < 0.01 for both) (Table 1). To gain more insight into the characteristics of each cluster, we compared the raw mean scores for the 5 NPSI dimensions across clusters. There were significant differences between clusters in the NPSI dimensions for deep pain, paroxysmal pain, and paresthesia/dysesthesia (Fig. 1B, Supplementary Table 2). Of note, the cluster 2, “paroxysmal pain”, was characterized by significantly higher scores in the dimensions for paroxysmal pain (electric shocks, stabbing) and paresthesia/dysesthesia but by lower scores in the dimension for spontaneous deep pain. The cluster 3. “spontaneous pain”, showed the opposite pattern of cluster 2 with above average superficial and deep pain and lower scores in paroxysmal and evoked pain. Finally, the cluster 1, “evoked pain”, was characterized by above average evoked pain and represented a mixed pattern of the other 2 clusters in the remaining dimensions (i.e., above average deep pain and paroxysmal pain but below average paresthesia/dysesthesia). Most of these characteristic neuropathic pain phenotypes were also reproduced in the features of the painDETECT questionnaire (Supplementary Table 3). Pain attacks with or without persistent pain were the predominant features in Clusters 1 and 2, whereas the opposite was true in Cluster 3. Patients with higher scores for electric shock-like sensations (indicative of paroxysmal pain) were also significantly overrepresented in Clusters 1 and 2 compared with Cluster 3.

### Overall effects of scrambler therapy

The primary analysis showed a significant reduction in the pain NRS scores over 2 weeks of treatment (percentage change from baseline at the end of treatment, least squares mean − 15%; 95% CI − 28% to − 2.4%; p < 0.001; Table 2). The mean score for the BPI interference subdimension was significantly improved with scrambler therapy (p = 0.022 for the effect of time), whereas the composite score for the BPI pain subdimension (mean of the 4 pain items) did not change significantly (Table 2). In post-hoc analysis of each BPI item, scrambler therapy resulted in significantly lower scores in terms of worst pain, average pain, general activity, mood, and sleep, but not in the other items (Supplementary Table 4).

**Table 2:** Efficacy of scrambler therapy.

	All (n = 27)	P-value	Cluster 1 (n = 8)	Cluster 2 (n = 10)	Cluster 3 (n = 9)	P-value
Pain NRS scores (percent change from baseline to day 10)	− 15 (− 28, − 2.4)	< 0.01	− 18 (− 36, 0)	− 23 (− 40, − 6.8)	− 3.7 (− 21, 13)	< 0.01
**BPI pain scores**						
Baseline	6.3 (5.2 7.4)	0.11	4.8 (3.3, 6.4)	6.4 (5.0, 7.8)	7.5 (6.1, 9.0)	0.17
2 weeks	5.6 (4.5, 6.7)		4.0 (2.4, 5.4)	5.2 (3.8, 6.6)	7.5 (6.1, 9.0)	
4 weeks	5.8 (4.7, 6.9)		3.7 (2.2, 5.3)	6.1 (4.7, 7.5)	7.5 (6.1, 9.0)	
**BPI interference scores**						
Baseline	5.7 (4.2, 7.2)	0.02	4,6 (2.6, 6.7)	5.4 (3.6, 7.3)	7.1 (5.2, 9.0)	0.07
2 weeks	4.6 (3.1, 6.1)		2.9 (0.9, 5.0)	4.5 (2.6, 6.4)	6.3 (4.4, 8.2)	
4 weeks	4.8 (3.3, 6.3)		2.5 (0.5, 4.6)	5.3 (3.5, 7.2)	6.5 (4.6, 8.4)	

### Differential response to scrambler therapy by neuropathic pain phenotypes

**Figure 2:**
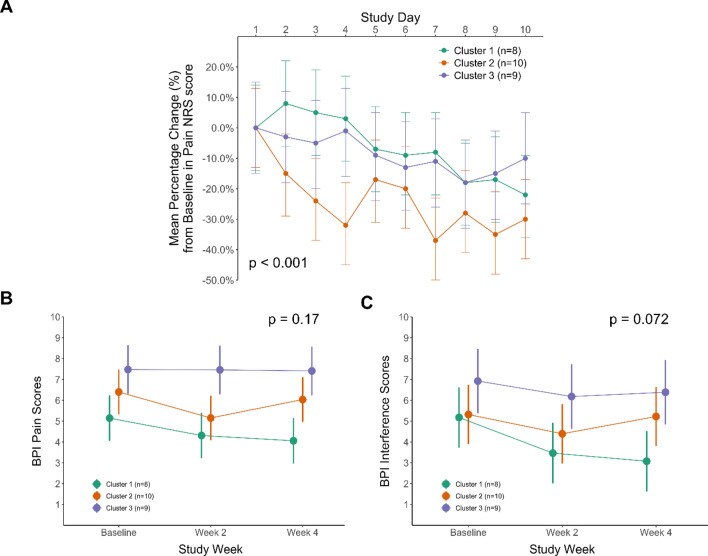
Percentage Changes from Baseline in Scores of the Pain Numerical Rating Scale (NRS) by Cluster (**A**), and the Absolute Changes in Mean Scores of BPI-SF Pain and Interference Subdimension by Cluster (**B**). The effect of scrambler therapy was analyzed using linear mixed effects model (LMM) with fixed effects for time, cluster, etiology, sex, use of anticonvulsants and the interaction terms (time × cluster and time × etiology) and random intercept for individual participants. Points and Bars represent least square means and 95% confidence intervals, respectively.

In a linear mixed-effects model analysis, there was a significant effect of the time × cluster interaction term (p = 0.003; Table 2; Fig. 2A) on the pain NRS scores, indicating a differential response to scrambler therapy across clusters. The composite score of the BPI pain subdimensions did not change differentially by cluster (Fig. 2B). However, the changes in the mean scores of the BPI interference subdimension showed a trend of different treatment responses by cluster (p = 0.072, effect of time × cluster interaction; Fig. 2C). In contrast, there was no significant effect of the time × etiology interaction term on the scores of the pain NRS or the BPI pain/interference subdimensions (Supplemental Figure 4).

Estimates of the mean percentage changes (for the pain NRS scores) and the mean absolute changes (for the mean scores of BPI pain and interference subdimensions) are given by the least-squares means (95% confidence intervals) in the linear mixed effects model. As fixed effects, we entered time (study day or week), cluster, etiology, sex, use of anticonvulsants and the interactions between of day and cluster and between day and etiology into the model. As random effects, the model assumed different intercept per subject. *P*-values were obtained by the likelihood ratio tests of the full LMM model against the model without the effect of time (for the overall effect) and without the interaction term between time and cluster (for the differential effect by cluster).

## Discussion

This study showed that 2 weeks of scrambler therapy resulted in a significant decrease in the scores of the pain NRS and BPI interference subdimension in patients with drug-resistant neuropathic pain of various etiologies. A post-hoc cluster analysis of the 10 NPSI item scores partitioned the patients into 3 subgroups with distinct neuropathic pain phenotypes: cluster 1 (“evoked pain”), cluster 2 (“paroxysmal pain”), cluster 3 (“spontaneous pain”). The treatment outcomes were significantly different across clusters, with a trend of more favorable outcomes in patients with preferentially paroxysmal pain rather than persistent pain.

Our results are in line with previous studies that support the benefits of scrambler therapy in drug-resistant neuropathic pain. While the efficacy of scrambler therapy in central neuropathic pain has rarely been investigated, we observed a favorable treatment outcome in the patients with spinal cord lesions of various etiologies including myelitis, ischemia, vascular malformation and subacute combined degeneration. In keeping with our results, a recent sham-controlled study involving 22 patients with neuromyelitis optica spectrum disorder showed that scrambler therapy can improve pain, depression, and anxiety^38^. However, the number of patients was too small to draw a firm conclusion, and the benefits of scrambler therapy for central neuropathic pain deserve further investigations with a larger number of subjects.

Whereas previous studies have reported 30% to 90% mean pain reductions, we noticed a rather modest efficacy in this study (22% mean reduction in the pain NRS scores)^13,15,17,39–43^. The discrepancy might be attributed to several reasons. First, most participants in our study had severe drug-resistant neuropathic pain (74% of patients had scores of 7 or greater in the pain NRS). Second, operator’s experience with scrambler therapy was somewhat limited, although a pre-trained operator strictly followed the established protocol as described and the whole process was supervised by an experienced technician and expert neurologist as well. Third, the vast majority of participants (93%) were receiving anticonvulsants prior to and during this study. It was claimed that the use of anticonvulsants could reduce the effects of scrambler therapy by interfering with action potential propagation^11,13,43^. Lastly, we could not exclude a reporting bias. While excellent outcomes have been reported mostly by retrospective case series or prospective trials with no-treatment control^13,15,42–44^, one sham-controlled, double-blind, randomized trial has shown a negative result^45^.

One of the interesting findings in our study is the differential response to scrambler therapy according to neuropathic pain phenotypes. While progress has recently been made in defining neuropathic pain phenotypes by analyzing pain-related symptom or sensory profiles, the results of cluster analysis in our study corroborate the findings of previous reports on phenotype-mechanism correlations^20,25,27,46–49^. We used the Pearson correlation distance as a dissimilarity measure for clustering, which we believe could better capture the distinct pattern of neuropathic pain phenotype than the conventional measure of Euclidean distance. In previous phenotyping studies using the Euclidean distance as a dissimilarity measure, the overall intensity of pain, rather than the distinct pattern of phenotype, accounted for most of the differences between clusters^21,50^. In contrast, the three patient subgroups in our study were similar in the overall pain severity but showed distinct characteristics in the neuropathic pain symptom profiles. Of note, the treatment outcomes were different across the clusters, raising the possibility of differential response to scrambler therapy depending on neuropathic pain phenotypes. For instance, Cluster 2, where the effect of scrambler therapy was most pronounced, was characterized by predominant paroxysmal pain and paresthesia but minimal persistent deep pain. Paroxysmal pain can be linked mechanistically to high-frequency bursts from damaged Aβ fibers, while the modest deep pain may reflect the relatively preserved C-fibers^46–49,51–55^. Provided that scrambler therapy delivers synthetic non-pain signals through C-fibers, it can be expected that the patients with denervated C-fibers are less likely to respond to the treatment^11^.

This study is an open-label trial and lacks a sham control. Other limitations include the small number of participants with heterogenous etiologies that may preclude a robust clustering. It also should be pointed out that our approach to phenotyping was based on the symptom profiles of NPSI and the questionnaire does not reflect sensory signs or negative sensory symptoms. As such, our approach may not capture the “sensory loss” phenotype which is one of the three clusters derived from QST profiles^20^. Because we did not perform QST, however, it was not possible to compare our clusters with those based on QST. Nonetheless, given the diverse manifestations of neuropathic pain, it would be interesting to compare or combine these two approaches to identify the most relevant aspects of neuropathic pain phenotype^22,28–31^. To the best of our knowledge, this study is the first to show the therapeutic implications of neuropathic pain phenotyping for scrambler therapy. Further studies with a larger number of patients and in diverse etiologies are needed to better define the pathophysiologically relevant pain phenotypes in a variety of neuropathic pain conditions, as well as to understand the mechanisms of action of scrambler therapy.

## Supplementary Information

Supplementary Information.

## Data Availability

The data that support the findings of this study are available from the corresponding author upon reasonable request.
