# Supplementary material for: Differential response to scrambler therapy by neuropathic pain phenotypes
Source: Sci Rep. 2021 May 12;11:10148. doi: 10.1038/s41598-021-89667-6 (PMC8115242; doi:10.1038/s41598-021-89667-6)

Differential Response to Scrambler Therapy by Neuropathic Pain Phenotypes

Young Gi Min[1], Hyun Seok Baek[1], Kyoung-Min Lee[1,2], Yoon-Ho Hong[2,3*]

[1]Department of Neurology, Seoul National University Hospital, Seoul, Korea

[2]Department of Neurology, Seoul National University College of Medicine, Seoul, Korea

[3]Department of Neurology, Seoul Metropolitan Government-Seoul National University Boramae Medical Center, Seoul, Korea

Corresponding author: Yoon-Ho Hong, Department of Neurology, Seoul Metropolitan Government-Seoul National University Boramae Medical Center and Seoul National University College of Medicine, 20 Boramaero-5-Gil, Dongjak-Gu, Seoul, 07061, Korea. E-mail: nrhong@gmail.com, Telephone: +82-2-870-2474, Fax: +82-2-831-2826

Supplementary materials

Supplementary Table 1. Validity Indices with Proposed Number of Clusters. A detailed description of each validity index can be found elsewhere (Charrad, 2014)

Validity index	Number of clusters proposed	Index value	Validity index	Number of clusters proposed	Index value
KL	3	2.1279	Beale	2	1.3515
CH	2	3.948	Ratkowsky	3	0.2693
Hartigan	3	1.4486	Ball	3	46.6012
CCC	2	-3.1346	PtBiserial	4	0.5722
Scott	4	64.1211	Gap	2	-1.5496
Marriot	4	2.75725E+11	Frey	1	NA
TrCovW	3	155.5083	McClain	2	0.7013
TraceW	3	12.0621	Gamma	5	0.7638
Friedman	4	11.3957	Gplus	5	7.2934
Rubin	3	-0.0494	Tau	3	52.3989
Cindex	5	0.3774	Dunn	4	0.3174
DB	5	1.6863	SDindex	5	1.0424
Silhouette	3	0.313	SDbw	5	0.5612
Duda	2	0.8235			
PseudoT2	2	3.2154			

Supplementary Table 2. Baseline Summary of the Neuropathic Pain Symptom Inventory (NPSI) by Cluster
Data are expressed as mean (SD) and number (%) as appropriate.
P-values are obtained using ANOVA for continuous variables and Fisher's exact test for the categorical variables.

	Cluster 1 (n=8)	Cluster 2 (n=10)	Cluster 3 (n=9)	P-value
NPSI subdimension				
Superficial pain	3.9 (3.4)	5.3 (3.9)	5.1 (4.0)	0.7
Deep pain	6.1 (2.2)	0.8 (1.0)	4.1 (3.8)	0.00079
Paroxysmal pain	4.0 (3.9)	6.7 (2.7)	2.3 (3.9)	0.034
Evoked pain	4.5 (2.6)	2.8 (2.2)	2.9 (2.5)	0.31
Paresthesia/Dysesthesia	3.8 (1.8)	6.3 (2.1)	5.3 (2.3)	0.055
Duration of spontaneous pain (superficial and/or deep, hours)				0.94
24	6	8	7	
8-12	1	0	1	
4-7	1	0	0	
1-3	0	1	0	
<1	0	1	1	
Frequency of paroxysmal pain (over the last 24 hours)				0.011
>20	2	2	0	
11-20	0	1	2	
6-10	2	0	0	
1-5	2	7	2	
0	2	0	5	

Supplementary Table 3. Baseline Summary of the painDETECT by Cluster
*Pain scores graded by 11-point numeric scale were categorized into weak (0-3), moderate (4-6) and strong (7-10) groups.
**Pain scores originally graded by 0-5 grades (never, 0; hardly noticed, 1; slightly, 2; moderately, 3; strongly, 4; very strongly, 5) were grouped into weak (0-1), moderate (2-3) and strong (4-5) categories.
P-values are obtained using Fisher's exact test.

	Cluster 1 (n=8)	Cluster 2 (n=10)	Cluster 3 (n=9)	P-value
Pain*				
Current	2:6:0	1:7:2	0:4:5	0.07
Maximum (4 weeks)	0:5:3	0:4:6	0:1:8	0.11
Average (4 weeks)	0:8:0	0:7:3	0:3:6	0.015
Pain course pattern				0.047
Persistent pain with slight fluctuations	4	1	6	
Persistent pain with pain attacks	1	4	0	
Pain attacks without pain between them	2	1	0	
Pain attacks with pain between them	1	4	3	
Radiating pain				0.77
Yes	6	7	5	
No	2	3	4	
Gradation of pain**				
Burning sensation	2:3:3	4:4:2	2:4:3	0.92
Tingling sensation	1:4:3	0:3:7	1:3:5	0.61
Pain by light touch	4:1:3	7:1:2	6:2:1	0.76
Electric shock-like pain	4:3:1	1:3:6	7:0:2	0.013
Pain on cold/heat stimulation	2:1:5	4:3:3	7:2:0	0.06
Numbness	1:2:5	1:1:8	0:1:8	0.72
Pain by slight pressure	3:1:4	6:3:1	2:4:3	0.27

Supplementary Table 4. Summary statistics of BPI-SF Item Scores.
Estimates of the mean absolute changes are given by the least-squares means (95% confidence intervals) in the linear mixed effects model with fixed effects for time and random intercepts for individual participants. *P*-values were obtained by the likelihood ratio tests of the full LMM model against the model without the effect of time.

	Baseline	Week 2	Week 4	P-values
Pain worst	7.7 (6.9, 8.5)	6.7 (6, 7.5)	6.7 (6, 7.5)	0.01
Pain least	5.1 (4.2, 6)	4.6 (3.7, 5.5)	5.1 (4.2, 6)	0.14
Pain average	6.4 (5.7, 7.2)	5.7 (5, 6.5)	5.9 (5.1, 6.6)	0.03
Pain current	6 (5.2, 6.8)	5.4 (4.6, 6.2)	5.5 (4.7, 6.3)	0.22
General activity	6.5 (5.4, 7.6)	5.6 (4.5 6.7)	5.7 (4.6, 6.7)	0.05
Mood	7 (5.9, 8)	5.4 (4.4, 6.5)	6 (5, 7)	<0.01
Walking	4.7 (3.5, 6)	4 (2.8, 5.2)	4.1 (2.9, 5.3)	0.07
Work	5.9 (4.9, 6.9)	5.1 (4.1, 6.1)	5.1 (4.2, 6.1)	0.10
Relations with others	4.6 (3.3, 5.9)	4 (2.7, 5.3)	4.5 (3.2, 5.8)	0.32
Sleep	5.7 (4.5 7)	4.5 (3.3, 5.7)	5.1 (3.9, 6.3)	0.02
Enjoyment of life	6 (4.9, 7.2)	5.4 (4.3, 6.5)	5.6 (4.5, 6.7)	0.36

Supplementary Figure 1. Intervention Protocol

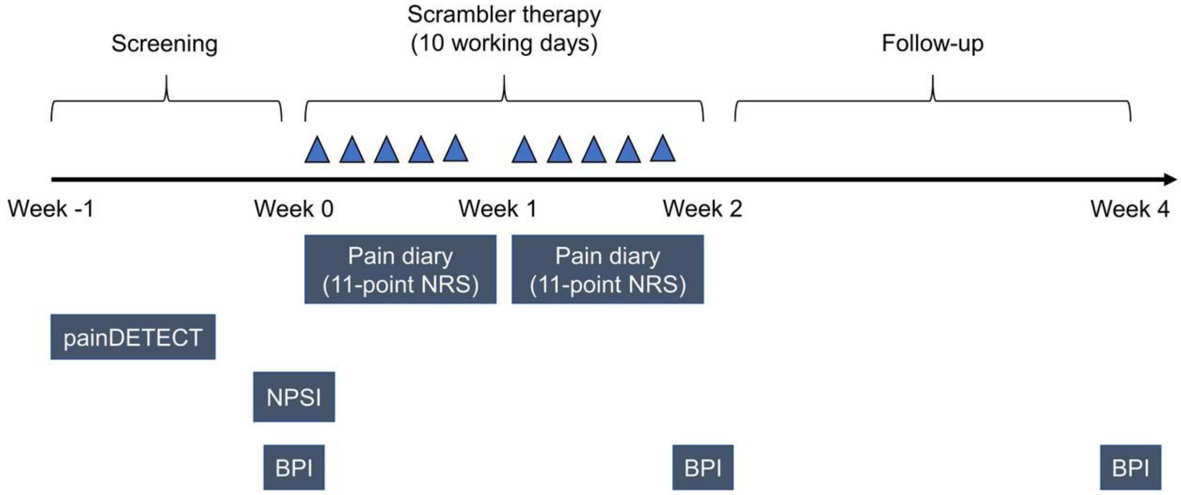

Supplementary Figure 2. Consort diagram of patient disposition

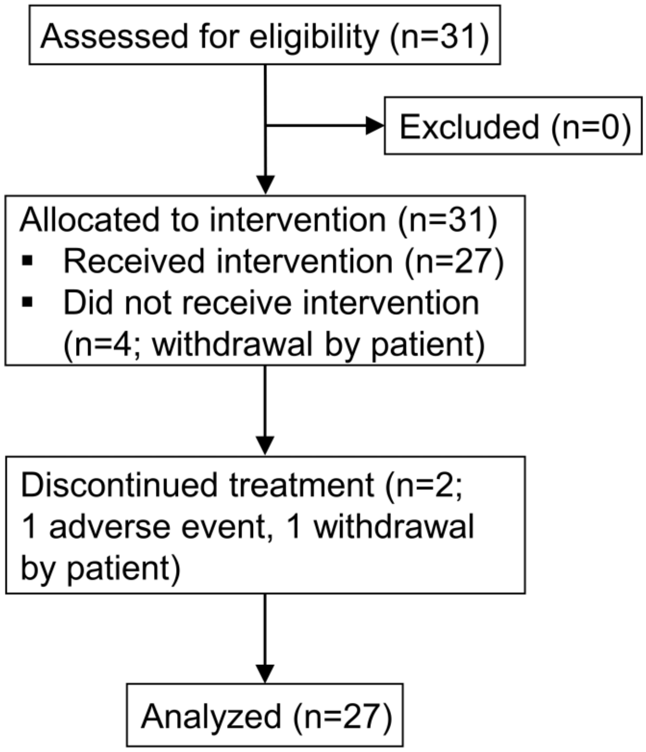

Supplementary Figure 3. Overview of Consensus Clustering

The consensus clustering was conducted with 80% patient resampling and a maximum K of 5. Iterative resampling was performed 1000 times using agglomerative hierarchical clustering algorithm on Pearson correlation distances. The left graph shows a heatmap of the consensus matrix for K=3 in which patients are represented as both rows and columns. Consensus values in the matrix range from 0 (never clustered together) to 1 (always clustered together) which are marked by white to dark blue colors. Cluster memberships (1, 2, 3) are marked by colored rectangles between the dendrogram and heatmap. The right graph shows the cumulative distribution function (CDF) of the consensus matrix for each K (cluster counts of 2, 3, 4, 5). The consensus CDF graph demonstrates a marked increase of the area under the curve for K=3 compared to K=2 and no appreciable increase with larger K, implying the optimal stability of clustering with K=3.

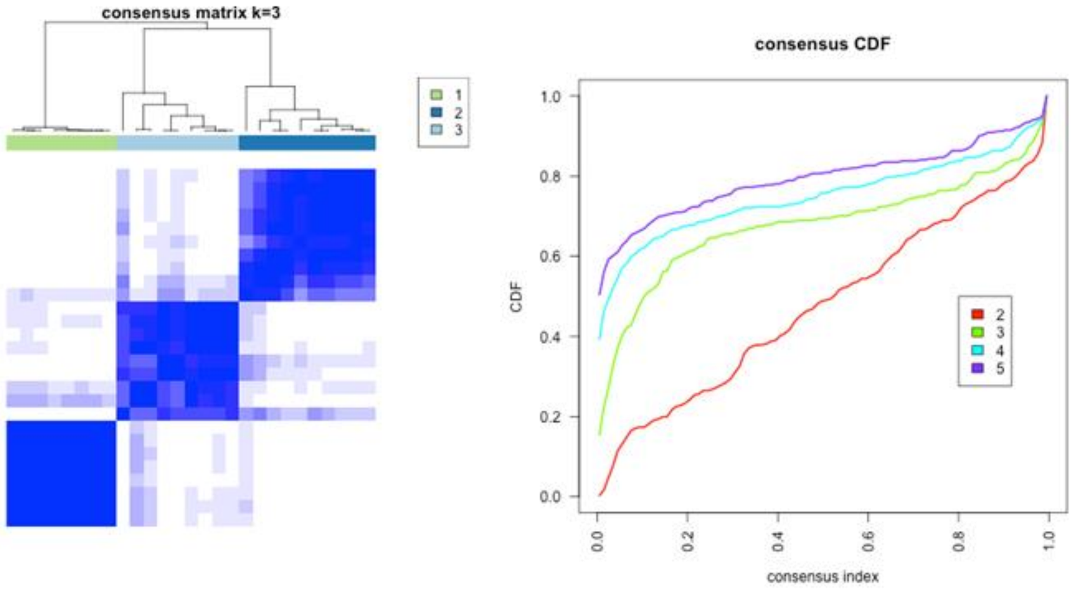

Supplementary Figure 4. Percentage Changes from Baseline in Scores of the Pain Numerical Rating Scale (NRS) by Etiology (A), and the Absolute Changes in Mean Scores of BPI-SF Pain and Interference Subdimension by Etiology (B). The effect of scrambler therapy was analyzed using linear mixed effects model (LMM) with fixed effects for time, cluster, etiology, sex, use of anticonvulsants and the interaction terms (time × cluster and time × etiology) and random intercept for individual participants. Points and Bars represent least square means and 95% confidence intervals, respectively.

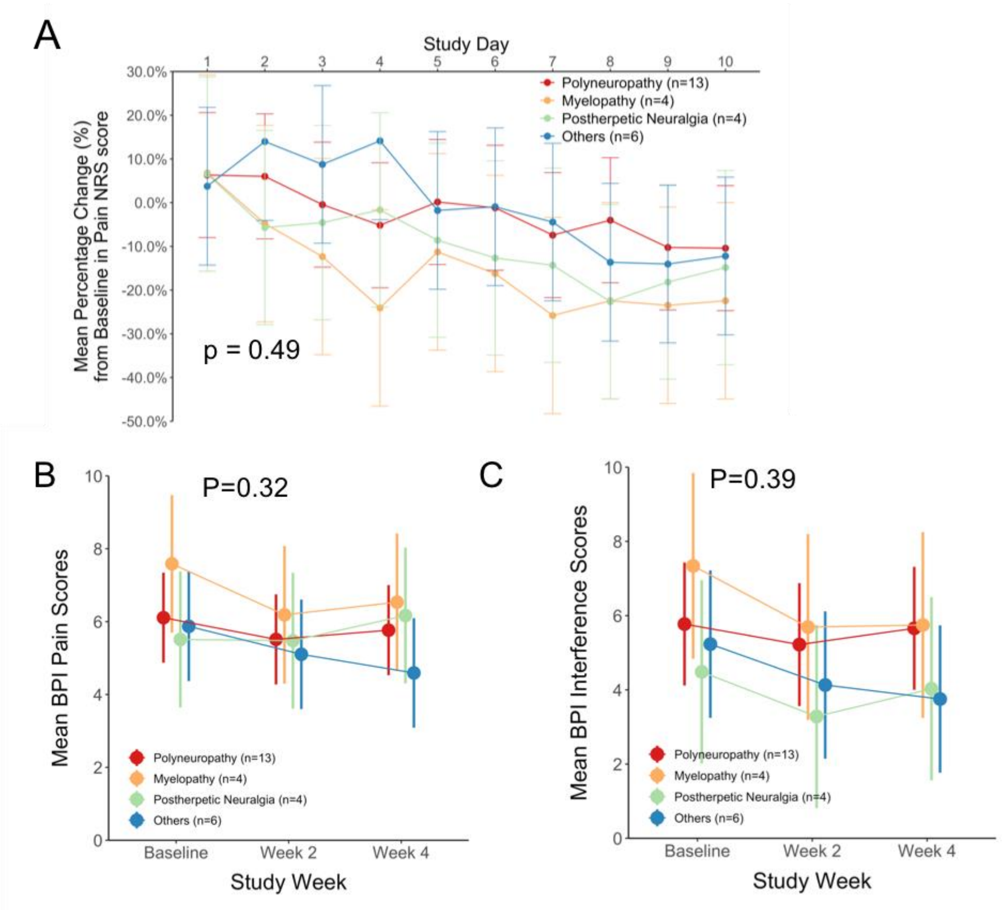

Supplement: Supplementary file 1 — Supplementary Information. [file 41598_2021_89667_MOESM1_ESM.pdf]
